# Supplementary material for: Analysis of PPARγ Signaling Activity in Psoriasis
Source: Int J Mol Sci. 2021 Aug 10;22(16):8603. doi: 10.3390/ijms22168603 (PMC8395241; doi:10.3390/ijms22168603)
Supplement: Supplementary file 1 [file ijms-22-08603-s001.zip › Supplemental materials_Analysis of PPARg signaling activity in psoriasis/Pathway models/Models images and html files/PPARG inhibits phenotypes assotiated with psoriasis/142837.html]

cell division


# Cell Process cell division

|  |  |
| --- | --- |
| URN | urn:agi-gocellproc:0051301 |
| Total Entities | 78 |
| Connectivity | 5597 |
| Name | cell division |
| ObjectType | Cell Process |

---

|  |  |
| --- | --- |
| ChildConcepts | progenitor cell renewal |
|  | mitosis |
|  | meiosis |
|  | telomere shortening |
|  | chromosome segregation |
|  | nuclear division |
|  | cell budding |
|  | asymmetric cytokinesis |
|  | stem cell renewal |
|  | schizogony |
|  | embryonic cleavage |
|  | endothelial cell division |
|  | lymphocyte division |
|  | CD4+ T-cell division |
|  | stem cell division |
|  | hematopoietic cell division |
|  | haematopoietic stem cell division |
|  | mast cell division |
|  | myeloid cell division |
|  | B-cell division |
|  | memory T-cell division |
|  | erythroid cell division |
|  | macrophage division |
|  | monocyte division |
|  | natural killer cell division |
|  | microglia division |
|  | mononuclear cell division |
|  | T-cell division |
|  | CD8+ T-cell division |
|  | fibroblast division |
|  | central memory T-cell division |
|  | blood platelet division |
|  | alveolar macrophage division |
|  | naive T-cell division |
|  | germinal center B-cell division |
|  | CD8+ memory T-cell division |
|  | dendritic cell division |
|  | activated T-cell division |
|  | immunocompetent cell division |
|  | erythroid precursor cell division |
|  | erythroblast division |
|  | leukocyte division |
|  | cancer stem cell division |
|  | regulatory T-cell division |
|  | Th17 cell division |
|  | umbilical vein endothelial cell division |
|  | kidney mesangial cell division |
|  | capillary endothelial cell division |
|  | lung fibroblast division |
|  | Kuppfer cell division |
|  | colony forming cell division |
|  | plasmablast division |
|  | immune system cell division |
|  | CD4+ memory T-cell division |
|  | myofibroblast division |
|  | plasma cell division |
|  | activated CD8+ T-cell division |
|  | helper T-cell division |
|  | bone marrow stromal cell division |
|  | peripheral lymphocyte division |
|  | phagocyte division |
|  | bone marrow macrophage division |
|  | bone marrow stem cell division |
|  | B-lymphoid precursor cell division |
|  | thymocyte division |
|  | effector T-cell division |
|  | bone marrow derived mesenchymal stem cell division |
|  | memory B-cell division |
|  | cytokine-induced killer cell division |
|  | peripheral blood stem cell division |
|  | plasmacytoid dendritic cell division |
|  | brain capillary endothelial cell division |
|  | activated CD4+ T-cell division |
|  | granulocyte division |
|  | myeloid progenitor cell division |
|  | Th2 cell division |
|  | lymphokine-activated killer cell division |
|  | blast cell division |

---

|  |  |
| --- | --- |
| Pathway | PPARG inhibits phenotypes assotiated with psoriasis |
|  | Possible Interplay between SOX11, CCND1 and EZH2 in Mantle Cell Lymphoma |

---

|  |  |
| --- | --- |
| MedScan ID | 4203581 |

---

|  |  |
| --- | --- |
| Alias | cell multiplications |
|  | cell replications |
|  | cell replication |
|  | divisions of the cell |
|  | cellular replications |
|  | cell cleavage |
|  | cell divisions |
|  | division of the cells |
|  | division of the cell |
|  | divisions of the cells |
|  | cellular divisions |
|  | cellular replication |
|  | cell division |
|  | cell multiplication |
|  | cellular division |
|  | cell cleavages |

---

|  |  |
| --- | --- |
| GO ID | 0051301 |

---
